# Impact of family integrated care on infants’ clinical outcomes in two children’s hospitals in China: a pre-post intervention study

**DOI:** 10.1186/s13052-018-0506-9

**Published:** 2018-06-05

**Authors:** Shi-wen He, Yue-e Xiong, Li-hui Zhu, Bo Lv, Xi-rong Gao, Hua Xiong, Huan Wang, Hua-rong Shi, Jos M. Latour

**Affiliations:** 1 Nursing Department, Hunan Children’s Hospital, Ziyuan Rd, Changsha, 410007 Hunan Province China; 2 Hunan University of Traditional Chinese Medicine, Xueshi Rd, Changsha, Hunan China; 3 Division of Neonatal Medicine, Hunan Children’s Hospital, Ziyuan Rd, Changsha, 41007 Hunan Province China; 4 Nursing Department, Maternal and Child Health Hospital of Guiyang Province, Ruijinnan Rd, Guiyang, Guizhou China; 5 School of Nursing and Midwifery, Faculty of Health and Human Sciences, University of Plymouth, Plymouth, UK

**Keywords:** Bronchopulmonary dysplasia, Clinical outcome, Family integrated care, Family centered care, Intensive care, Neonatology, Parents, Preterm infants

## Abstract

**Background:**

Most Neonatal Intensive Care Units (NICUs) in China have restricted visiting policies for parents. This also implicates that parents are not involved in the care of their infant. Family Integrated Care (FIC), empowering parents in direct care delivery and decisions, is becoming the standard in NICUs in many countries and can improve quality-of-life and health outcomes of preterm infants. The aim of this study was to evaluate the impact of a FIC intervention on the clinical outcomes of preterm infants with Bronchopulmonary Dysplasia (BPD).

**Methods:**

A pre-post intervention study was conducted at NICUs in two Chinese children’s hospitals. Infants with BPD were included: pre-intervention group (*n* = 134) from December 2015 to September 2016, post-intervention (FIC) group (*n* = 115) and their parents from October 2016 to June 2017. NICU nurses were trained between July and September 2016 to deliver the FIC intervention, including parent education and support. Parents had to be present and care for their infant minimal three hours a day. The infants’ outcome measures were length-of-stay, breastfeeding, weight gain, respiratory and oxygen support, and parent hospital expenses.

**Results:**

Compared with control group (*n* = 134), the FIC group (*n* = 115) had significantly increased breastfeeding rates (83% versus 71%, *p* = 0.030), breastfeeding time (31 days versus 19 days, *p* < 0.001), enteral nutrition time (50 days versus 34 days, *p* < 0.001), weight gain (29 g/day versus 23 g/day, *p* = 0.002), and significantly lower respiratory support time (16 days versus 25 days, *p* < 0.001). Oxygen Exposure Time decreased but not significant (39 days versus 41 days *p* = 0.393). Parents hospital expenses in local Chinese RMB currency was not significant (84 K versus 88 K, *p* = 0.391).

**Conclusion:**

The results of our study suggests that FIC is feasible in two Chinese NICUs and might improve clinical outcomes of preterm infants with BPD. Further research is needed to include all infants admitted to NICUs and should include parent reported outcome measures. Our study may help other NICUs with limited parental access to implement FIC to enhance parental empowerment and involvement in the care of their infant.

## Background

In 2016, the World Health Organization (WHO) reported that there are about 15 million preterm babies born each year worldwide and the number is still rising. At present, the number of preterm infants in China ranks second in the world [1]. Complications from preterm births are the most common cause of death among five-year-old children. About 70% of perinatal diseases occur in preterm infants. The incidence of cerebral palsy in preterm infants is 70 to 80 times that of full-term infants, and the rate of cognitive impairment is also significantly higher in preterm infants than in full-term infants [2]. In addition, it was found that up to 800 million U.S. dollars (USD) in costs are incurred due to preterm infants each year [3], leading to a significantly greater economic burden on their families. These expenses decrease as the number of gestational weeks and birth weight increases [4]. At the same time, the prolonged hospital stays of preterm infants have a serious impact on familial, social and medical resources [5].

The WHO proposed several relevant measures to improve the quality-of-life and health of preterm infants. These include regularly updating the clinical guidance for management of pregnant women at risk of preterm birth or mothers of preterm infants and guidelines for preterm infant nursing professionals in terms of kangaroo care, feeding of low birth weight infants, treatment of infectious and respiratory diseases and family-centered care [1]. Kangaroo care [6] and family-centered care [7] encourage greater involvement of parents in giving direct care to their infants in the Neonatal Intensive Care Unit (NICU). For a greater involvement of parents in the care of their infant the NICUs need to be liberal in the visiting policies. Most NICUs in China have restricted visiting policies and parents have limited involvement in care. In contrast, NICUs in higher resources countries welcome parents without restrictions. However, evidence suggests that in some European countries the visiting policies vary. A survey published 10 years ago among 175 NICUs in eight European countries identified that the majority of the participating NICUs in Italy (*n* = 35) and Spain (*n* = 22) had limited visiting hours while one third of the French NICU (*n* = 45) did not have unrestricted visiting hours for parents [8]. More recently, Raiskila and colleagues documented significant differences between 11 NICUs in six European countries in physical parent–infant closeness and presence [9]. Welcoming parents without restrictions and supporting them in the care of preterm infants might improve the quality of care. Therefore, the clinical staff should strive for family-centered care interventions and integrate the parents in care and decision-making processes in the NICU.

Family Integrated Care (FIC) is an approach that allows parents to provide non-medical routine care for their preterm infant during NICU hospitalization after they have been provided with education and guidance by well-trained neonatal nurses [10]. When parents are permitted to be closer to their infant and become more involved in providing care with the support of nurses, a good relationship between parents and nurses is essential. This can result in higher parent satisfaction with care and the parents become more confident in their parental roles [11]. A study in a Dutch NICU showed that parents rated their involvement in care as very important and they were more satisfied with care if they receive information about their infant and participate in the development of the treatment plan [12, 13]. Thus, nurses and doctors must recognize and acknowledge the wishes of parents based on their social and cultural needs [14–16].

The involvement of parents in providing care to infants at the NICU may affect the stability of preterm infants and the incidence of disease [17]. Bronchopulmonary dysplasia (BPD) is still a common disease in premature infants [18–20]. Due to immature lung development and few alveolar surfactants, premature infants are prone to respiratory distress syndrome and BPD. The use of mechanical ventilatory strategies may influence the incidence of BPD. Furthermore, in China, many tertiary NICUs are in a children’s hospital requiring transport of critically ill infants from a maternity hospital to these NICUs. Infants with delayed transport to a tertiary NICU had a higher incidence of BPD (57%) compared to transport within 24 h [21]. Given the increased emphasis of early parental education in NICUs, the role of parents can become important in the early stages of a NICU admission and might improve infant’s health outcomes. However, research on the effects of FIC on infants with BPD is sparse and overall the effects of FIC on the general NICU population remains limited. Therefore, the aim of the study was to evaluate the impact of a FIC intervention on the clinical outcomes of preterm infants with BPD.

## Methods

### Design

This study used a pre-post intervention design and was conducted between December 2015 and June 2017.

### Settings

Hunan Children’s Hospital is the largest child care center in Hunan province. The NICU is a tertiary neonatology center with 60 beds and admitting around 85 BPD infants every year. Guiyang Children’s Hospital is a specialist hospital and the NICU is the referral center of neonates in Guizhou province. The NICU has 70 beds and annually around 73 infants with BPD are treated. Parents in both NICUs are not allowed to visit their infants. Communication with parents is done via the NICU doctors three times a week and parents are able to see their infant via a video-connection.

### Participants and recruitment

Infants were eligible when they met the following four criteria:1) weight ≥ 1800 g with ventilatory support or weight ≥ 1500 g with non-invasive oxygen support; 2) stable hemodynamic condition; 3) some form of respiratory support or oxygen therapy is still required at the corrected gestational age of 36 weeks (FiO_2_ ≥ 0.3); 4) parents agreed to follow a training and take care of their child for at least three hours per day.

Exclusion criteria were: 1) severe congenital anomalies or respiratory deformities such as laryngeal cartilage dysplasia; 2) surgery; 3) receiving palliative care; 4) brain damage; 5) parents have serious social problems or language issues; 6) expected discharge within one week.

### Interventions

Infants with BPD hospitalized at both NICUs between December 2015 and September 2016 were the standard care group, and those admitted between October 2016 and June 2017 were the intervention group. The NICU nurses were trained from July to September 2016 to effectively deliver the intervention to parents of infants in the intervention group.

In the standard care group, parents were asked to follow the routine caring model and comply with the hospital rules and regulations. The parents were not allowed to be involved in the treatment and care of their infants except for visits by means of video connection every Monday, Wednesday, and Friday.

The FIC intervention required parents to accept the FIC training from qualified nurses before they entered the NICU. The training items included: 1) Hand hygiene: Parents were instructed to wash their hands under running water in 7 steps, and informed of the times to wash hands, such as before entering the NICU and before feeding. 2) Neonatal feeding: Parents were encouraged to breastfeed their infant and communicate at the same time to allow the infant to become familiar with the mother, feel loved and relieve anxiety. 3) Neonatal contact: Parents were told to touch the infant in the sequence of head and face, chest, abdomen, limbs, hands and feet then back with moderate to intense force and rub large muscle groups. 4) Patting on the back of the infant: Parents were instructed to smoothly pat the back of their infant with hollow hands from the outside to the inside and from top to the bottom 30 min before a meal or two hours after. 5) Involvement of care: Parents were encouraged to bath the infant, change diapers, and perform other basic care.

Parents in the FIC group were involved in non-medical care of their infant for at least three hours a day between 10:00 and 16:00. In these periods parents were encouraged to talk with their infant and play music.

### Data collection

Each NICU appointed one nurse (member of the FIC research group) to collect data from the medical records. Length-of-stay was the number of NICU days because all infants are discharged home directly from the NICU. Oxygen exposure time was defined as the number of days the infant received any form of oxygen support. Respiratory support time was defined as the number of days with invasive mechanical ventilatory support. The definition of breastfeeding time was the time the infant received partial or full breastfeeding per day [22]. The enteral nutrition time included the days of breastfeeding and formula. Weight gain was calculated by the formula: weight at discharge minus weight at admission divided by the length of NICU stay. BPD categorized in recovery, incomplete recovery, and death after NICU discharge. Readmission within one month after NICU discharge and hospital expenses were also collected. Hospital expenses were retrieved from the medical records and were related to the treatment and care expenses the parents had to pay.

### Data analysis

The statistical analysis was performed using SPSS19.0 software. Descriptive statistical methods were used to analyze the data. The t-test and χ^2^ test were used to compare the clinical outcomes between the FIC group and the standard care group. A *p*-value of < 0.05 indicates statistical significance.

### Ethical considerations

The study protocol was approved by the Institutional Review Board of Hunan Children’s Hospital (HCHLL-2015-33). The data of the standard care group were retrieved from the medical records and made anonymous to include in the study analysis. Therefore, a signed consent form was waived. Parents in the FIC group received an invitation letter and information about the study. A member of the FIC research group also verbally explained the study. Parents could withdraw participation without reason. A signed informed consent was required and collected.

## Results

**Fig. 1:**
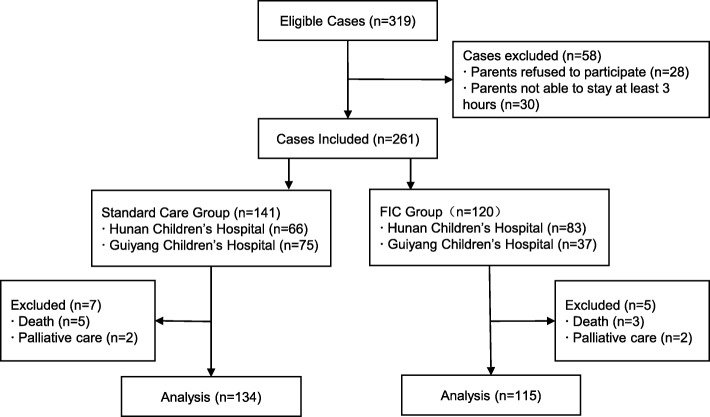
Flowchart of study participants. Legend. FIC=Family Integrated care

A total of 319 cases were eligible, of which 58 cases were excluded because parents did not want to participate or were unable to stay for the required hours per day. Among the remaining 261 cases, 13 infants died or were in palliative care. Included in the final analysis were 115 infants in the FIC group and 134 in the standard care group (Fig. 1).

### Characteristics of infants and parents

A total of 249 infants were included in the study, of which 168 were male. The mean gestational age and birth weight of infants in the FIC group were significantly lower than in the standard care group (*P* < 0.05). Also, father’s education was higher in the FIC group (Table 1).

**Table 1:** Infant and Parent Characteristics

Characteristics	FIC (*n* = 115)	Control (*n* = 134)	*P* value
Male (*n*, %)	75 (65.2)	93 (69.4)	0.482^1^
Female (*n*, %)	40 (34.8)	41 (30.6)	
Gestational age; weeks (mean, SD)	29.9 (1.8)	30.79 (2.0)	<0.001^2^
Birth weight; gram (mean, SD)	1352.6 (267.5)	1441.2 (376.8)	0.021^2^
Father’s age; years (mean, SD)	33 (5.8)	32 (6.3)	0.401^2^
Father’s education (*n*, %)			
Below high school	31 (27.0)	61 (45.5)	0.004^1^
High school	32 (27.8)	36 (26.9)	
Above high school	52 (45.2)	37 (27.6)	
Mother’s age; years (mean, SD)	30.(4.8)	29 (4.9)	0.087^2^
Mother’s education (n, %)			
Below high school	38 (33.0)	55 (41.0)	0.293^1^
High school	26 (22.7)	32 (23.9)	
Above high school	51 (44.3)	47 (35.1)	

^1^Chi-square test (χ^2^); ^2^Student t-test; *FIC* Family Integrated Care, *SD* Standard Deviation

### Clinical outcomes of infants with BPD

Table 2 shows the clinical outcomes of infants with BPD. There were significant differences (P < 0.05) in respiratory support time (invasive or non-invasive ventilator support), breastfeeding rate, breastfeeding time, enteral nutrition time and weight gain rate between the infants of the FIC group and standard care group. No significant differences (*P* > 0.05) were found in length-of-stay, hospitalization expenses, oxygen exposure time and BPD outcome.

**Table 2:** Infants’ Clinical Outcomes FIC and Control Group

Outcomes		FIC *n* = 115	Control *n* = 134	*P* value
Length-Of-Stay; days (mean, SD)		52 (10.5)	49 (20.2)	0.084^2^
Hospital expenses (RMB, mean, SD)		84,409 (27,766.2)	87,602 (37,343.5)	0.391^2^
Oxygen exposure time; days (mean, SD)		39 (14.9)	41 (13.8)	0.393^2^
Respiratory support time; days (mean, SD)		16 (10.8)	25 (13.0)	< 0.001^2^
Breastfeeding	Yes (%)	95 (82.6)	95 (70.9)	0.030^1^
	No (%)	20 (17.4)	39 (29.1)	
Breastfeeding time; days (mean, SD)		31 (20.2)	19 (19.5)	< 0.001^2^
Enteral nutrition time; days (mean, SD)		50 (15.9)	34 (22.9)	< 0.001^2^
Weight gain rate; grams/day (mean, SD)		28.5 (14.6)	23.3 (9.9)	0.002^2^
BPD outcome	Complete recovery (*n*, %)	81 (70.4)	86 (64.2)	0.422^1^
	Incomplete recovery (*n*, %)	34 (29.6)	45 (33.6)	
	Death (*n*, %)	0	3 (2.2)	
Re-admission within 1 Month	Yes (*n*, %)	14 (12.2)	21 (15.7)	0.492^1^
	No (*n*, %)	101 (87.8)	113 (84.3)	

^1^Chi-square test (χ^2^); ^2^ Student t-test; *FIC* Family Integrated Care, *SD* Standard Deviation, *BPD* Bronchopulmonary Disease, *RMB* RenMinBi (Chinese Yuan)

## Discussion

The FIC model was implemented in the NICUs of two Chinese children’s hospitals to test the feasibility of this model. Our study provides evidence that FIC might improve the clinical outcomes of infants with BPD and could provide a reference for further utilization of FIC in other children’s hospitals in China. A survey of 129 pediatric nurses in Hunan Province about the knowledge, attitudes and behaviors of FIC before and after a training course revealed that most nurses (74%) were willing to deliver the FIC model regardless of the training [23]. Consequently, with the results of this intervention study we now participate in a larger multi-center RCT to test FIC in a larger NICU population [24].

The FIC model allows the parents of infants with BPD to participate in the care and provides them with training to better understand the methods and benefits of breastfeeding. The breastfeeding rate and time in our study was significantly higher in infants in the FIC group than in the standard care group. Verma and colleagues also confirmed in their study that providing training to parents and involving parents in care (*n* = 148) increases the breastfeeding rates to 80% [25].

We found that the respiratory support time for infants with BPD in the FIC group was significantly reduced. Ortenstrand, et al. [26] also demonstrated that family involvement in care significantly reduced the number of infants with moderate to severe BPD from 6% in the standard care group to 1.6% in the family care group. Thus, FIC might suggest that parents’ involvement in care might have a positive effect on the infants’ health and outcomes.

Although the length-of-stay in our study did not show significant improvements, Melnyk et al. [7] and Bhutta et al. [27] demonstrated that allowing parents to care for their infant can reduce length-of-stay. However, Welch et al. [28] argued that although the average length-of-stay of infants in a FIC model was 3.4 days less than that for infants under routine care and the median length-of-stay was 4 days less, there was no statistical significance. Our results indicated no significant difference even though the length-of-stay of infants in the FIC group was longer than the control group, which may be associated with the significantly lower gestational age of infants in the FIC group than the standard care group. The respiratory support time of infants in our FIC group was significantly lower than the standard care group, but the oxygen exposure time and length-of-stay of the FIC group were significantly higher than the standard care group, resulting in higher average hospitalization expenses of the FIC group. Therefore, the impact of the FIC model on length-of-stay and hospitalization expenses needs to be further studied.

Our study is subject to some limitations. Firstly, parents only stay for a minimum of three hours a day, which is short and fragmented. A recent study used the inclusion criteria of a minimum of eight hours and even more time with the increase of the demands of the infant for breastfeeding [10]. The clinical outcomes of the infants showed a significant positive weight gain and a significant increase in breastfeeding. We acknowledge that the length of time of parents taking care of their infant could have influenced our results. Secondly, private rooms were not available for parents and their infants to spend time together due to lack of space in the NICU. Therefore, we placed a screen at the infant’s bedside to provide privacy for parents to interact with their infant. A systematic review identified the benefits of single room designed NICUs and conclude that single family room NICUs might decrease length-of-stay and reduce readmissions [29]. Thirdly, we observed a substantial imbalance of the number of cases in the FIC intervention group between the two hospitals; 37 infants were included from Guiyang Children’s Hospital in the FIC group compared to 83 infants from Hunan Children’s Hospital (*n* = 83). It was unclear how the differences occurred. A reason could be the termination of the one-child policy beginning 2016 which might have led to differences in birth rate in the two provinces. The differences in proportion of infants from the two hospitals between the pre and post-intervention groups could represent a bias for the results and a larger study in the future is needed to test the effect of a FIC intervention. Finally, the Chinese tradition of “zuo yuezi” (sitting the month) means that mothers are expected to rest indoors and avoid heavy physical activity for one full month after giving birth. Therefore, several infants were cared for by their fathers but after discharge were then cared for by the mothers at home. Therefore, specific information about discharge planning that might have influenced the readmission rate could not be shared with the mothers.

## Conclusion

Our study suggests that the FIC model is feasible in NICUs and might result in significant improvements of infants’ clinical outcomes such as weight gain, breastfeeding time, breastfeeding rate and respiratory support time. Therefore, this study might be supportive to all NICUs who consider implementing the involvement and empowerment of parents in the care and decision making of their infant. However, further studies are needed to explore the benefits of FIC in infants and parent reported outcomes.

## Abbreviations

BPD: Broncho-pulmonary Disease
FIC: Family Integrated Care
NICU: Neonatal Intensive Care Unit
RMB: RenMinBi (Chinese Yuan)
SD: mean and standard deviation
USD: U.S. dollar
WHO: the World Health Organization
